# Molecular Bases and Specificity behind the Activation of the Immune System OAS/RNAse L Pathway by Viral RNA

**DOI:** 10.3390/v16081246

**Published:** 2024-08-02

**Authors:** Emma Jung-Rodriguez, Florent Barbault, Emmanuelle Bignon, Antonio Monari

**Affiliations:** 1 Université Paris Cité and CNR, ITODYS, F-75006 Paris, France; emma.jung-rodriguez7@etu.univ-lorraine.fr (E.J.-R.); florent.barbault@u-paris.fr (F.B.); 2 Université de Lorraine and CNRS, LPCT UMR 7019, F-54000 Nancy, France; emmanuelle.bignon@univ-lorraine.fr

**Keywords:** innate immune system, oligoadenylate synthase, RNA viruses, molecular dynamics, free energy profiles

## Abstract

The first line of defense against invading pathogens usually relies on innate immune systems. In this context, the recognition of exogenous RNA structures is primordial to fight, notably, against RNA viruses. One of the most efficient immune response pathways is based on the sensing of RNA double helical motifs by the oligoadenylate synthase (OAS) proteins, which in turn triggers the activity of RNase L and, thus, cleaves cellular and viral RNA. In this contribution, by using long-range molecular dynamics simulations, complemented with enhanced sampling techniques, we elucidate the structural features leading to the activation of OAS by interaction with a model double-strand RNA oligomer mimicking a viral RNA. We characterize the allosteric regulation induced by the nucleic acid leading to the population of the active form of the protein. Furthermore, we also identify the free energy profile connected to the active vs. inactive conformational transitions in the presence and absence of RNA. Finally, the role of two RNA mutations, identified as able to downregulate OAS activation, in shaping the protein/nucleic acid interface and the conformational landscape of OAS is also analyzed.

## 1. Introduction

RNA viruses are a class of pathogens which represent a significant threat due to their capability to induce epidemic or pandemic outbreaks [1]. Indeed, the World Health Organization (WHO) has classed different RNA viruses among the most serious emerging infectious diseases [2]. Stunning evidence of the potential disruptive threat of RNA viruses has come to light with the emergence of the COVID-19 pandemic [3,4,5,6,7], which started at the end of 2019 in Wuhan, China, and spread to virtually every continent by the spring of 2020, pushing authorities to implement severe social distancing measures, including full lockdowns. COVID-19 infection is caused by a β-stranded positive RNA virus belonging to the *coronaviridae* family [3], which has been named SARS-CoV-2, and shares homology with other potentially lethal coronaviruses such as SARS-CoV [8], which appeared in 2003, and MERS-CoV, which emerged in 2013 [9]. The RNA viruses encompass other extremely virulent pathogens, such as hemorrhagic fever viruses (Ebola), flaviruses, and arboviruses including Zika [10,11], West Nile [12], and Chikungunya viruses [13]. Interestingly, since the latter pathogens exploit insects, particularly mosquitos, as vectors, their worldwide diffusion has increased significantly as a consequence of global warming [14], which enables the colonization of temperate regions by vectors originally developing in tropical areas. Furthermore, common seasonal influenza, which is correlated each year with a non-negligible mortality rate and was the origin of serious pandemic events in the 20th century, is also caused by a rapidly mutating genome-segmented RNA virus [15,16].

The frontline of the immune system’s defense against invasion by RNA viruses in vertebrates usually relies on the innate immune system and, in particular, on its capacity to sense the presence of exogenous genetic material, such as specific RNA sequences and structural motifs [17,18]. The antiviral innate immune response is usually characterized by the activation of pro-inflammatory signaling, as well as the production of interferons and cytokines [19], thus developing an unfit microenvironment and inducing apoptosis or senescence of infected cells. The combination of these responses is aimed at slowing the virus’s spread, limiting its diffusion. Different innate immune system pathways have been characterized in recent years. These involve the cyclic GMP–AMP synthase/stimulator of interferon genes (cGAS/STING) pathway [20], which is activated by the presence of exogenous DNA, and to a less extent RNA [21], and is mediated by the signaling exerted by small cyclic nucleotides. Interestingly, overactivation of the STING protein has been correlated to the insurgence of serious COVID-19 outcomes, and, in particular, to the cytokine storm which is one of the main causes of morbidity of SARS-CoV-2 [22]. Yet, the main reaction to the presence of pathogen RNA is the one mediated by 2′,5′-oligoadenylate synthase (OAS) and RNAse L proteins [23,24,25]. In this pathway, the catalytic activity of OAS is triggered by its interaction with double-strand RNA fragments and leads to the production of short 2′-5′-oligoadenylate oligomers. The latter, in turn, are used as signals to mediate further activation of the RNAse L endonucleases which cleave, non-specifically, single-strand RNA. The cleavage of both viral and cellular RNA, including messenger RNA, leads to apoptosis of the cell and thus stops viral reproduction and diffusion [24]. The role and efficiency of the OAS pathway in the defense against viral aggression has been particularly underlined in the case of SARS-CoV-2, in which human haplotypes of Neanderthal descendance, which preferentially colocalize with the reproduction compartment of the virus, have been correlated to milder or even asymptomatic infections [26].

Usually, three main variants of OAS are encountered in vertebrates [27]. The smaller OAS1 presents only one catalytic unit and is particularly efficient in recognizing short double-strand RNA oligomers [28]. Two catalytic cores are bridged together in the case of the OAS2 variant, leading to the recognition of medium-length RNA oligomers, while three units are bridged in the case of OAS3, thus allowing interactions with longer strands. Interestingly, only two of the catalytic centers in OAS3 are active, while the third is not reactive but participates in the recognition of viral RNA.

**Figure 1:**
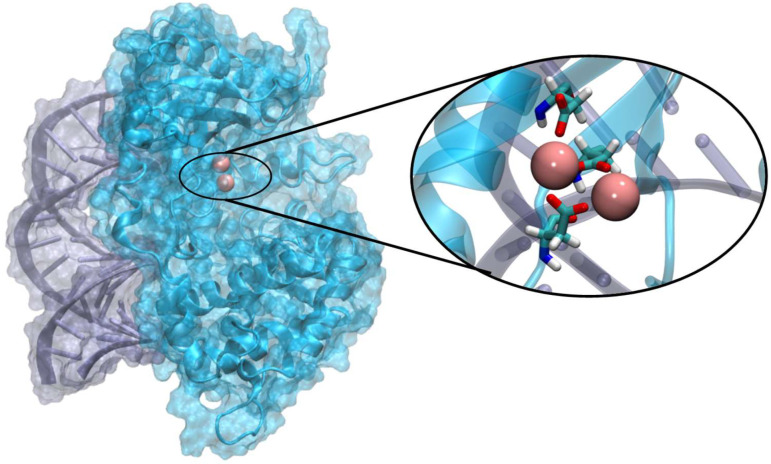
Representative snapshot showing the structure of the human OAS1 protein (in cyan) interacting with model RNA double strand (in purple). In the inlay, there is a zoomed-in element of the active site showing the Mg_2_^4+^ cluster complexed by aspartate ligands.

From a biochemical and biophysical point of view, as shown in Figure 1 for OAS1 [29,30,31], the binding of viral RNA involves a large recognition area which is spatially distant from the catalytic centers, which presents a [Mg_2_]^4+^ cluster, similarly to the majority of polymerases and nuclease enzymes. Therefore, the activation of OAS should involve RNA-driven allosteric regulation of the catalytic activity, which enables activation of the pathway only in the presence of exogenous genetic material. Recently, it has been shown that the human OAS1 presents a strong selectivity toward the 5′-untranslated region (5’-UTR) of the SARS-CoV-2 genome and, in particular, its first stem loop motif (SL1) [32,33]. The affinity of OAS for this motif could also explain its specific activation since 5’-UTR and SL1, in particular, are highly conserved regions and exert an important regulatory role in the viral cycle, in particular concerning RNA translation and replication [34,35]. Similar structural regions involving stem loops and presenting regulatory activity are also found in other RNA viruses, such as Dengue [36], West Nile [37], or Zika [38], and are specifically recognized by OAS1 [39]. In the past, by using long-range classical molecular dynamics (MD) simulations, we have shown that the while the specific recognition of SARS-CoV-2 SL1 is mainly driven by sequence-specific interactions with the extruded nucleobases [32], the tertiary arrangement of the West Nile SL1 offers structural-based specific interaction patterns [39]. The crystal structure of OAS1 in the active form [29], i.e., complexed with model double-stranded RNA (in the following sections referred as a HOLO structure), has been obtained, as well as its inactive counterpart in the absence of RNA (APO form), revealing important structural differences. In addition, as shown by Donovan et al. [29], site-specific mutations of the RNA sequence downregulate the activation of OAS1. Yet, the molecular and atomistic factors dictating the allosteric regulation of OAS1 remain partially elusive. Therefore, in this contribution, we scrutinize the human OAS1 in active and inactive form and the interplay with RNA in modulating the allosteric conformational transition of OAS1. In addition, we also analyze the structural effects of RNA point mutations [29] and the effects of RNA in modulating the free energy profile of the active vs. inactive transition of OAS1.

## 2. Materials and Methods

The initial structure of the human OAS1 enzyme in the active form was retrieved from the PDB data base (id 4IG8) [29]. The structure also included the presence of an 18-mer RNA double strand of sequence 5′-GGCUUUUGACCUUUAUGC-3′ which was maintained (HOLO system). Missing residues were added using the Swiss-Model online server [40]. No structure of the human OAS in inactive APO form could be found in the pdb data base. Therefore, we constructed an APO form of OAS1, simply removing the RNA double-strand from the structure of PDB 4IG8; this system will be further referred to as APO1. In addition, we constructed a second model (APO2) using the crystal structure of the porcine OAS1 in its APO form (PDB id: 4RWQ) [25], which shares 74% homology with the human protein, as a template for the homology model, again using the Swiss-model server. To simulate the effects of the mutation of the RNA in the affinity towards OAS1, we also constructed two further systems by manually mutating the base pairs 17 (GC17AU) and 18 (GC18AU), referred to as HOLO17 and HOLO18 in the following, respectively.

All the systems were soaked in a cubic water box enforcing a 9 Å buffer, using the amber tleap utilities [41] and neutralized with the minimum amount of K^+^ ions. The OAS1 protein was modeled using the amberff19SB force field [42], while RNA was described with the OL3 RNA force field [43] and water with the TIP3P model [44,45]. All the equilibrium MD simulations were performed in the isotherm and isobaric (NPT) ensemble at a temperature of 300 K and pressure of 1 atm, which were enforced using a Langevin thermostat [46] and barostat [47]. Particle Mesh Ewald (PME) summation with a cut-off of 9 Å were consistently used. The Hydrogen Mass Repartition (HMR) strategy [48] was used in combination with Rattle and Shake algorithms [49] to enable the use of a time step of 4 fs for integrating the Newton equations of motion. Prior to the production run, the system was optimized using a conjugated gradient algorithm and equilibrated and then thermalized by progressively removing constraints on the RNA and protein backbone atoms during three consecutive steps of 36 ns each. All the MD simulations were performed using the NAMD code [50,51] and were analyzed with VMD [52] and cpptraj [53] utilities.

In addition to equilibrium MD simulations, and to enforce the transition between the active and inactive conformations in the HOLO and APO systems, we performed enhanced sampling MD simulations. To this aim, the difference in the backbone root mean square distribution (ΔRMSD) between representative structures of the two conformations, obtained from the equilibrium MD of HOLO and APO2, respectively, was used as the collective variable. The free energy profile connecting the two conformations was obtained by the umbrella sampling (US) procedure using forces between 1.0 and 10.0 N. The suitability of the collective variable was previously examined performing Steered Molecular Dynamics (SMD), ensuring that the path was indeed connecting the two equilibrium conformations. Enhanced sampling was performed using NAMD coupled to the Colvar [54] utilities. The cavity was identified and quantified using the CASTpFold webserver [55].

## 3. Results

### 3.1. Equilibrium MD Simulation

**Figure 2:**
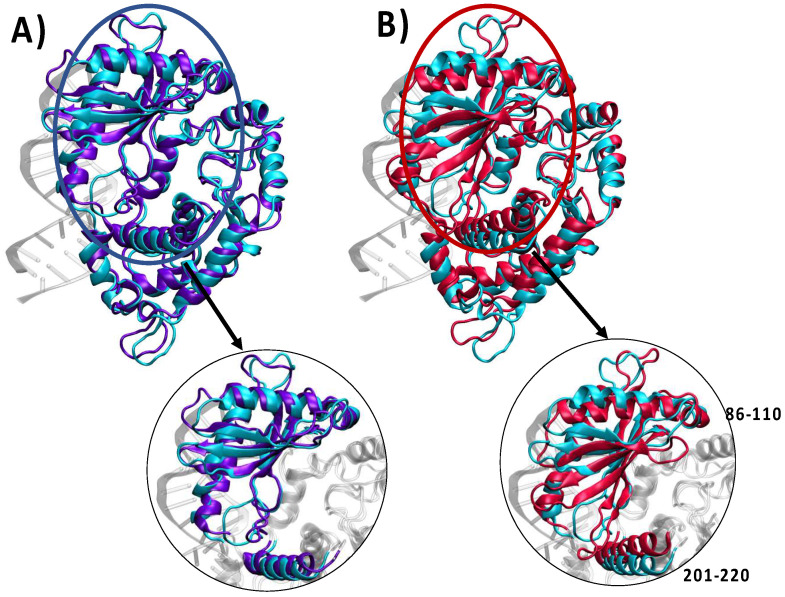
Superposition of representative snapshots issued from the MD simulation between the HOLO (cyan) and the APO1 (panel (**A**) in purple) and APO2 (panel (**B**) in red). A zoomed-in section of the region mostly affected by the conformational changes is also provided.

The MD simulations of both the HOLO and the reconstructed APO1 and APO2 models are stable and converged at the μs time-scale, as can be notably observed from the analysis of the time-evolution of the root mean square deviation (RMSD), which is reported in SI. In Figure 2, we report the superposition of the structures obtained for both APO models with the HOLO system. Of note, in the presence of double-strand RNA, and at least at the time-scale of our dynamics, the HOLO system experiences a remarkable conformational stability, presenting only very limited deviation from the crystal structure. As shown in Figure 2A, the APO1 structure, which has been constructed by simply removing the RNA double strand, is not able to recover the native structure and remains locked in a conformation, which presents an almost perfect superposition with the HOLO system. On the contrary, significant structural changes can be observed in the case of the APO2 system. This concerns, in particular, the position of the two α-helices involving residues 86–110 and 201–220, respectively, and the sliding and rotation of the β-sheet region. Interestingly, the most important global structural changes do not involve areas which are in direct contact with the RNA recognition interface, thus confirming a halosteric regulation of the activation of OAS1. Indeed, the changes observed may instead affect the accessibility of the active site, involving the Magnesium cluster catalytic center. This effect can be directly assessed by measuring the volume of the cavity encompassing the active center, which amounts to 2.6 and 2.0 nm^3^ for HOLO and APO2. Interestingly, and confirming the locking of this conformation in a metastable state, APO1 presents a value of the cavity volume absolutely comparable with the one of the original HOLO system. Our MD simulations are also in line with the results inferred by Donovan et al. [29] by analyzing the different crystal structures. Our results are then consistent in identifying an active conformation, which is populated in the HOLO structure upon interaction with RNA, and an inactive state which is characteristic of OAS1 at rest. In this respect, we may consider that APO1 should not be considered as a physically or biologically relevant state but instead as a computational artifact.

### 3.2. RNA-Induced Structural Reorganization

When switching to the analysis of the RNA-induced structural reorganization at a residue level, it was pointed out [29] that the allosteric propagation towards the active site was mainly driven by the basic residues K66 and R195, which should switch their position to optimize their interactions with E233.

**Figure 3:**
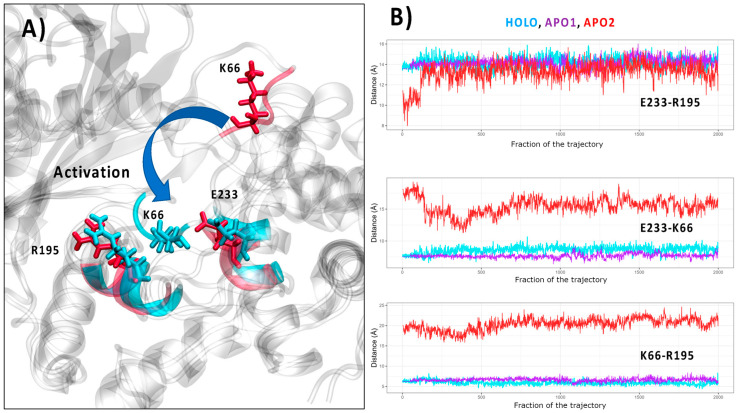
(**A**) Superposition of the HOLO (cyan) and APO2 (red) structures highlighting the position of the residues E223, K66, and R195 in the two structures. (**B**) Time evolution of the distances between the residues E233, R195, and K66 in HOLO (cyan), APO1 (purple), and APO2 (red).

As can be appreciated from Figure 3A, the transition between the HOLO and APO2 form involves a rather important rearrangement of this triad, in particular concerning the placement of K66. On the contrary, as reported in SI, no significant reorganization can be observed for APO1, once again pointing to the fact that this conformation is locked in a metastable state. Interestingly, from the time evolution of the residue–residue distance reported in Figure 3B, it can be appreciated that while K66 is isolated in the APO2 conformation, it develops a stable interaction with both the side chain of E223 and the backbone of R195 in the HOLO conformation. However, no direct interaction between R195 and E223 can be observed in either HOLO or APO1/APO2 simulations. Therefore, our results may suggest that, different to what was supposed from the analysis of the different crystal structures, the conformational transition between the active and inactive form does not involve switching between the positions of the two basic residues but rather a double role of K66 which bridges together E223 and R195, exploiting both electrostatic interactions with the side chains and hydrogen bond stabilization with the backbone. This observation is also confirmed considering the average distances between the centers of mass of the K66 and E223 residues in HOLO and APO2 conformations, which amount to 5.8 ± 1.0 and 17.1 ± 1.5 Å, respectively, while the average distance of the center of mass between K66 and R195 is 9.0 ± 1.0 and 23.7 ± 2.0 Å for the HOLO and APO2 conformations, respectively. The locking of K66 in the HOLO form may also be inferred from the variation in the root mean square fluctuation reported in SI (Figures S8 and S9), which shows a strong reduction in the flexibility of this residue upon recognition of the RNA.

### 3.3. Free Energy Profile for the Active/Inactive Transition

To better assess the thermodynamics and kinetics of the conformational transitions provoked by the interaction with RNA, we have used umbrella sampling-enhanced MD simulations to enforce the active/inactive conformational transition in HOLO and APO systems, i.e., in the presence and absence of RNA. The free energy profiles related to this transition are determined by sampling the path connecting the active (HOLO) and inactive (APO2) state, by considering the variation in the RMSD between the two equilibrium states.

**Figure 4:**
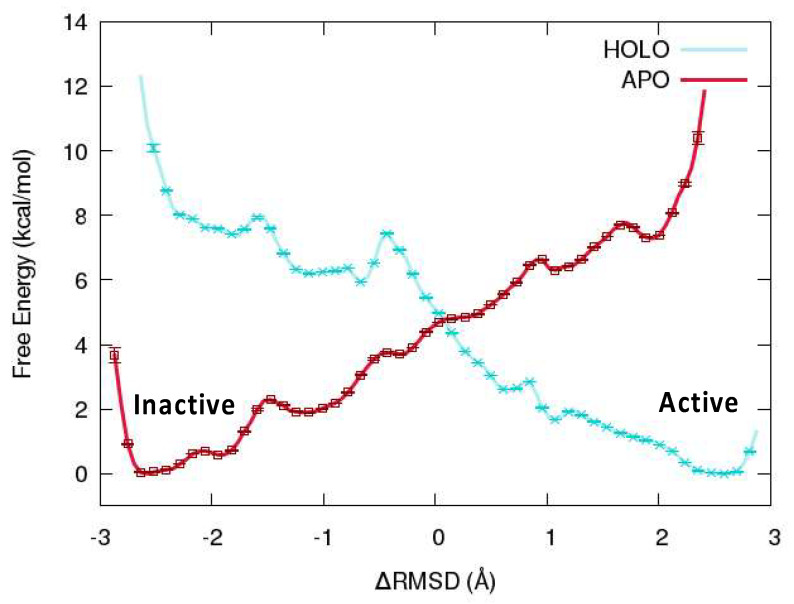
Potential of mean force for active/inactive transition in HOLO (cyan) and APO2 (red) systems obtained from umbrella sampling-enhanced MD simulations. Note that the inactive conformation ΔRMSD = −2.7 Å, i.e., the minimum free energy for the APO system, corresponds to the APO2 conformation.

The free energy profile and potential of mean force (PMF) are reported in Figure 4, from which it can be readily observed that the global minimum of the APO2 system corresponds to the inactive conformation (ΔRMSD = −2.6 Å). However, in the case of the HOLO system, the active conformation (ΔRMSD = +2.6 Å) is more favorable. Interestingly, the free energy increase when forcing the active conformation of the HOLO form (with RNA) towards the inactive one corresponds to about +12 kcal/mol. Conversely, the penalty of maintaining an active conformation in the absence of RNA amounts to about 10 kcal/mol. In both the HOLO and APO systems, the transition towards the most stable conformation proceeds rather smoothly, without the need to bypass significant free energy barriers (the highest one amounting to 2 kcal/mol). This result is consistent with the biological role of OAS1, which should be rapidly activated upon sensing the presence of viral RNA and should be readily inactivated in the absence of exogenous RNA. On the other hand, the fact that the inactive conformation was not recovered for the APO1 system should probably be due to the large inertia correlated to the extended conformational rearrangement. As a matter of fact, an equilibrium MD simulation in which the RNA double strand was manually docked onto APO2 system in inactive conformation has shown partial recovery of the active form (see ESI).

After considering the thermodynamics and kinetic factors determining the conformational equilibrium between the active and inactive form upon recruitment or release of double-strand RNA oligomers, we scrutinized in more detail the inherent factors stabilizing the protein/nucleic acid interface, hence favoring recognition.

**Figure 5:**
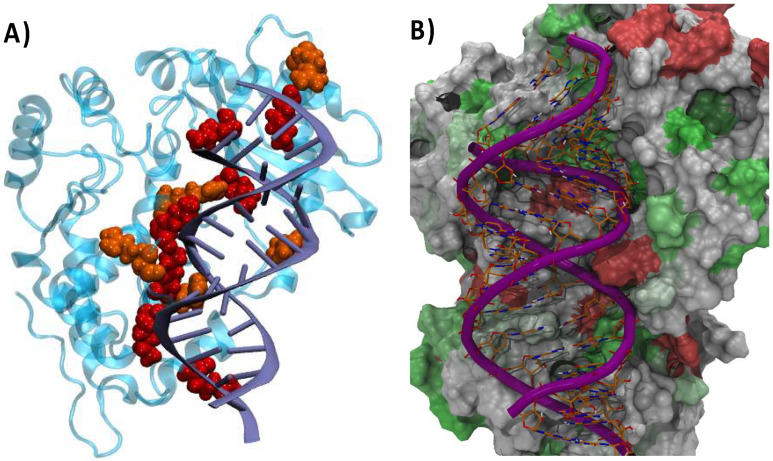
(**A**) The interaction interface between OAS1 and the model double-strand RNA model. The protein interfacial basic residues (lysine red and arginine orange) are highlighted in van der Waals representation. (**B**) A representation of the hydrophobicity of the OAS1 protein interacting with the RNA. The protein is rendered in surface representation. Residues colored in green are hydrophobic, in red are hydrophilic, and in white are amphiphilic.

In Figure 5A, we report a representative snapshot highlighting the interacting interface between OAS1 and our model RNA. Unsurprisingly, the interface presents a high density of basic, positively charged residues, mainly lysine and arginine. This accumulation of basic residues participates in creating an electrostatically positive channel which may efficiently accommodate the negatively charged RNA backbone. The presence of such a recognition motif is also widely common in proteins interacting with nucleic acid, including histones and polymerases. While the electrostatic interface clearly represents the main factor maintaining the RNA/OAS1 complex, it is important to underline that other non-covalent interactions take place and contribute to the stabilization of the interface. Interestingly, as shown in Figure 5B, we may also identify a cluster of hydrophobic residues at the center of the RNA/protein interface. These residues may reinforce the specificity of the recognition by developing favorable interactions with the nucleic acid nucleobases. Indeed, it has also been recently shown that in some cases the recognition of viral material by OAS1 may also be mediated by the interaction with extruded nucleobases [32].

In particular, the analysis of the MD simulation for the HOLO system has shown the establishment of some rather persistent hydrogen bonds also involving the peripheral nucleobases and the nearby protein residues. A summary of the different hydrogen bonds, together with their persistence along the MD simulation, are reported in Table 1. It is evident that in the original system the RNA/OAS1 interface is also stabilized by stable hydrogen bonds, which are also characterized by near ideal angles and distances between the donor and the acceptor. 

These results are also consistent with previous MD simulations devoted to the analysis of the specific recognition of SARS-CoV-2’s 5′-UTR domain, which has shown sequence-dependent additional stabilization by hydrogen bonds with extruded nucleobases [32].

### 3.4. Sequence Specificity of the RNA Recognition

**Figure 6:**
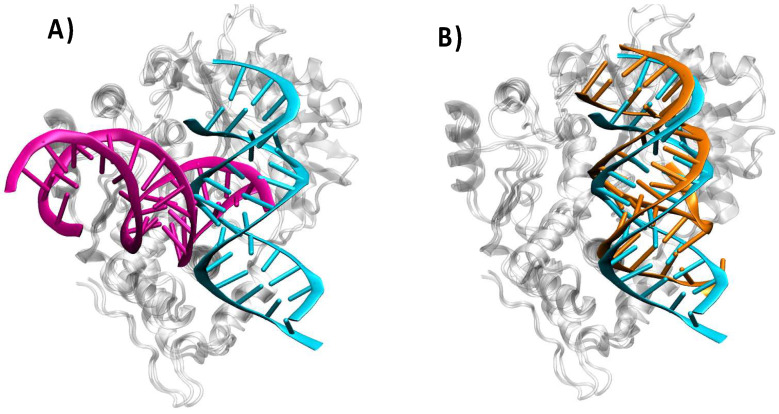
Representative structure of the HOLO17 ((**A**), magenta) and HOLO18 ((**B**), orange) mutants in complex with OAS1 and superposed to the original HOLO structure (in cyan).

It has recently been shown that two specific mutations in the double-strand RNA sequence, namely GC17AU (HOLO17) and GC18AU (HOLO18), correlate with a downregulation of the OAS1 activation [29]. From the results reported in Table 1, it appears evident that the hydrogen-bond network in the case of the mutated oligomers has been almost completely disrupted, with only one, relatively weak, hydrogen bond persisting for both mutants. Interestingly, the hydrogen bond interactions have also been lost in cases of nucleobases not directly involved in the two mutations, suggesting a subtly regulated tuning of the interfacial recognition. However, the sole observation of the native HB network perturbation masks a completely different structural organization between the two structures. Indeed, as shown in Figure 6, the recognition interface is completely lost in HOLO17. Instead, in the case of HOLO18, and despite the loss of hydrogen bond interactions, the protein/RNA interface is relatively well conserved, even if the activation of OAS1 catalytic activity is reduced. This evidence further confirms that OAS1 is indeed able to enforce a more specific recognition of RNA sequences, thanks notably to the establishment of specific hydrogen bond networks. This is again consistent with specific activation of the OAS/RNAse L pathway only in the presence of exogenous infections, thus avoiding overactivation of the innate immune system. It is also in line with our results evidencing the role of hydrogen bonding in leveraging specific recognition of SARS-CoV-2 genetic material [32].

## 4. Discussion and Conclusions

By using long-range MD simulations complemented with enhanced sampling and free energy methods, we have unraveled the molecular bases related to the subtle conformational equilibrium of OAS1 upon binding to double-stranded RNA. Indeed, we have confirmed the presence of an active and an inactive conformation, which differ by the relative orientation of α-helixes and β-sheets. The allosteric regulation, leveraging the population of the active conformation, leads to an enlargement of the catalytic site pocket. In addition, we have shown that the driving force for the population of the active conformation is about 12 kcal/mol and that the transition does not involve significant energy barriers. As concerns the RNA/protein interaction, we have shown that, in addition to the expected salt bridges and electrostatic interactions, an extended network of hydrogen bonds is present that stabilizes the interface and drives the transition towards the active form. Indeed, we have shown that in the case of activity loss—mutations involving the peripheral nucleobases of the double-stranded oligomer—the native hydrogen bond’s pattern is almost entirely disrupted. Interestingly, this situation may also lead to complete loss of the correct RNA positioning in the case of the GC17AU mutant. Our results, while confirming experimental observations and previous MD simulations, shed further light on the thermodynamics driving the activation of OAS1 and thus triggering the innate immune system upon recognition of exogenous genetic material. The role of hydrogen bonds, which lead to sequence-specific recognition of RNA strands and contribute to structural recognition, has also been clearly evidenced. Interestingly, the fact that both a double helical arrangement and the presence of sequence-specific interactions are necessary for proper activation of OAS1 should also minimize the possibility of activation of the immune system by recognition of endogenous RNA, thus leading to autoimmune disorders. However, this aspect does necessitate more specific studies in order to be fully addressed. The knowledge brought about by our work can help in understanding the factors modifying the immune system response and eventually in designing specific immunotherapy drugs aimed at viral or autoimmune diseases.

In the future, we plan to extend this study elucidating the thermodynamics and inactive/active transition of OAS1 taking into account its interaction with actual viral sequences such as the first stem loop in 54-UTR of SARS-CoV-2 or West Nile viruses.

## Figures and Tables

**Table 1:** The main hydrogen bond interactions take place along the MD simulation for the native HOLO and the mutated HOLO17 and HOLO18. The frequency of occurrence is given together with the average distance and angle calculated along the MD simulation. Residues belonging to RNA are preceded by an uncapitalized ‘r’ for clarity.

	H-Acceptor	H-Donor	Frequency %	Average Distance (Å)	Average Angle (°)
HOLO					
	V55	rG17	70.08	2.1 ± 0.5	141 ± 23
	S56	rG17	69.76	2.0 ± 0.2	155 ± 12
	V58	rA16	59.68	2.1 ± 0.3	140 ± 13
	Q200	rG8	56.85	2.8 ± 1.0	137 ± 27
	rU15	K60	68.71	2.0 ± 0.3	166 ± 8
	rA13	N31	65.73	2.1 ± 0.4	156 ± 15
	rU6	N27	59.92	3.1 ± 4.0	133 ± 40
	rU7	N27	55.89	2.6 ± 1.4	127 ± 45
	rA9	Q200	55.65	2.3 ± 0.8	134 ± 44
	rU7	T24	51.37	1.7 ± 3.0	132 ± 44
HOLO17					
	rG3	T203	55.00	7.3 ± 9.0	135 ± 42
HOLO18					
	V58	rA16	65.56	2.0 ± 0.3	141 ± 16

## Data Availability

Data are available upon reasonable request.

## Supplementary Materials

The following supporting information can be downloaded at: https://www.mdpi.com/article/10.3390/v16081246/s1, Details on the definition of the collective variable. Time evolution of the RMSD for the different systems studied (Figures S1–S4, S6 and S7). Superposition of the APO2 + RNA and HOLO system (Figure S5). RMSF for the HOLO and APO2 systems (Figure S8) and their difference (Figure S9). Distribution of the values of the collective variables over the umbrella sampling (Figure S10).
